# The complete mitochondrial genome of the hybrid of *Leiocassis longirostris* (♂)×*Pelteobagrus fulvidraco* (♀)

**DOI:** 10.1080/23802359.2017.1372711

**Published:** 2017-08-30

**Authors:** Guosong Zhang, Xia Liang, Jiajia Zhang, Jie Li, Shaowu Yin

**Affiliations:** a College of Life Sciences, Key Laboratory of Biodiversity and Biotechnology of Jiangsu Province, Nanjing Normal University, Nanjing, Jiangsu, China;; b Department of Life Science, Heze University, Heze, Shandong, China;; c Co-Innovation Center for Marine Bio-Industry Technology of Jiangsu Province, Lianyungang, Jiangsu, China

**Keywords:** *Pelteobagrus fulvidraco*; *Leiocassis longirostris*, mitogenome, hybrid

## Abstract

In this work, we reported the complete mitochondrial DNA sequence of the hybrid of *Leiocassis longirostris* (♂)×*Pelteobagrus fulvidraco* (♀), which was obtained by artificial hybridization. The total length of the mitochondrial genome is 16,527 bp, with the base composition of 30.86% for A, 25.56% for T, 28.20% for C, and 15.38% for G. It contains two ribosomal RNA genes, 13 protein-coding genes, 22 transfer RNA genes, and a major non-coding control region (D-loop region). The arrangement of these genes is the same as that found in the teleosts. All the protein initiation codons are ATG, except for COX1 that begins with GTG. The complete mitogenome of the hybrid provides an important data set for the study in mitochondrial inheritance mechanism. Both the termination-associated sequence and critical central conserved sequences (CSB-D, CSB-E, and CSB-F) were also detected.

*Pelteobagrus fulvidraco* and *Leiocassis longirostris* belong to the family Bagridae. The hybrid of *P. fulvidraco* (♀)×*L. longirostris* (♂) combines the desirable traits of *P. fulvidraco* (appearance) and *L. longirostris* (growth performance). Recently, farming scale of the hybrids has been gradually increased in Asia, suggesting a promise of a new variety for yellow catfish. There is no report of the complete genome of this hybrid. In the present study, parental fish of *P. fulvidraco* (♀, individual weight of over 100 g) and *L. longirostris* (♂, over 500 g) was obtained by north latitude 32titu and east longitude 118gitu. The hybrids were obtained by artificial hybridization on 1 June 2017 in Nanjing Fisheries Research Institute at Nanjing, Jiangsu Province, China and its tailfins were preserved in 95% alcohol. All DNA were extracted using phenol–chloroform extraction methods and stored at −80 °C.

All newly determined sequences from this study were deposited in GenBank database (accession number: MF583740). The total length of the hybrid mitochondrial DNA was 16,527 bp, which includes the 13 protein-coding genes, 22 tRNA genes, two rRNA genes and two non-coding regions: control region (D-loop region) and the origin of light-strand replication. All genes showed the typical gene arrangement conforming to the vertebrate consensus (Prosdocimi et al. 2012). The A + T content of the hybrid mitogenome is 56.42%, and the content is 30.86% for A, 25.56% for T, 28.20% for C, and 15.38% for G. A high A + T content indicates an obvious antiguanine bias commonly observed in fishes (Qiao et al. 2012). Except for eight tRNA (*Gln, Ala, Asn, Cys, Tyr, Ser, Glu*, and *Pro*) genes and one protein-coding gene (*ND6*), most of the genes were encoded on the heavy strand (H-strand). All genes displayed the typical gene arrangement conforming to the vertebrate consensus (Chen et al. 2012). In the 13 protein-coding genes of the hybrid mitochondrial genome, four overlaps are detected as shown in: *ATP8-ATP6, ATP6-COXIII, ND4-ND4L*, and *ND5-ND6* sharing 10, 1, 7, and 4 nucleotides, respectively. The overlap of the ATPase genes appears to be common in most vertebrate mitochondrial genome (7–10 bp) (Broughton et al. 2001). Among the 22 tRNA genes, three overlaps are found, i.e. *tRNA^Ile^-tRNA^Gln^, tRNA^Gln^-tRNA^Met^* and *tRNA^Thr^-tRNA^Pro^*. There are also two overlaps between *ND2* and *tRNA^Trp^* and between *ND3* and *tRNA^Arg^*. The non-coding control region (D-loop) of the hybrid was 888 bp, flanked by *tRNA^Pro^* and *tRNA^Phe^* genes (Figure 1).

**Figure 1.**
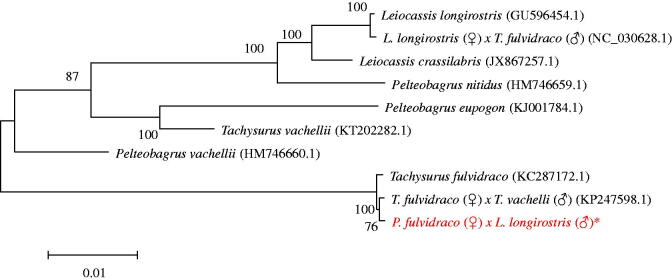
The evolutionary tree with 10 typical *Leiocassis* and *Pelteobagrus* family. *The hybrid of *P. fulvidraco* (♀)×*L. longirostris* (♂) (accession number: MF583740) in the position of the evolutionary tree. Numbers above branches are bootstrap values by 1000 replicates. The 10 typical Siluriformes species according to their classification can be divided into four families, represented by ‘}’. The Genbank accession numbers of the sequences for the other 20 kinds of fishes were used in the tree as follows: *L*. *crassilabris* (JX867257.1), *L*. *longirostris* (GU596454.1), *L*. *longirostris* (♀)×*T*. *fulvidraco* (♂) (NC_030628.1), *P. eupogon* (KJ001784.1), *P. vachellii* (HM746660.1), *P. nitidus* (HM746659.1), *Tachysurus fulvidraco* (KC287172.1), *T*. *fulvidraco*×*T*. *vachelli* (KP247598.1), *T*. *vachellii* (KT202282.1).

To determine the mitogenome nucleotide differences between the hybrid, *P. fulvidraco* (PF2012, NC_015888.1), *P. fulvidraco* (ECSFRI-PF01, NC_023767.1), and *L. longirostris* (NC_014586.1), we performed some pairwise analyses. The mitogenome nucleotide of the hybrid has a 99.85%, 99.78%, and 90.84% similarity with PF2012, ECSFRI-PF01 and *L. longirostris*. This result suggests that low level of mitogenome variation exists between the hybrid and *P. fulvidraco* (PF2012 and ECSFRI-PF01) (Liang et al. 2012; Wan et al. 2014). The conserved sequence blocks of CSB2 was identified in the positions 16,389–16,405 bp. The sequence of this region is 5′-AAACCCCCCTACCCCC-3′. The conserved sequence blocks of CSB1 and CSB3, the termination associated sequence and the critical central conserved sequences of CSB-D, CSB-E, and CSB-F occur at the same positions with *P. fulvidraco* (PF2012) (Liang et al. 2012).
